# Stress and headaches in university students during the COVID-19 pandemic

**DOI:** 10.1371/journal.pone.0288745

**Published:** 2023-11-22

**Authors:** Jenna Francisco, Faly Golshan, Todd G. Morrison, Marla Mickleborough

**Affiliations:** 1 Department of Psychology and Health Studies, College of Arts and Science, University of Saskatchewan, Saskatoon, Saskatchewan, Canada; 2 College of Medicine, University of Saskatchewan, Saskatoon, Canada; Universidad Privada San Juan Bautista, PERU

## Abstract

With regards to the relationship between mental and physical health conditions, some studies predict increased headache frequency as a result of exposure to stressful situations. Thus, the purpose of our study was to investigate whether headache characteristics among a sample of university students (*N* = 234) correlated with fear of COVID-19, stress and quality of life vis-à-vis the pandemic. We found statistically significant positive correlations between headache frequency and both general stress and quality of life. Further, results from a multiple regression analysis suggested that fear of COVID-19 did not account for incremental variance in headache intensity. Explanations for our key findings, limitations of our study, and future directions for post pandemic studies on headache experience are outlined.

## Introduction

Headaches are a predominant and sometimes debilitating pain experience that have diverse causes. While some headaches are a secondary disorder that might be resulting from head injury (such as post-concussion impact), a larger variety are found to be primary headache disorders including tension headaches and migraines [1]. According to the World Health Organization [2], headache disorders occur in approximately 50% of the adult population. The cause of headaches is varied and sometimes uncertain; however, both physiological and psychological factors have been identified as potential causal agents. Some of the most common environmental and physiological triggers for headaches include sensory (light or sound) stimuli, hormone imbalance, weather changes, certain medications or foods, and stress [3]. Stress is a biological and psychological response to demands or threats placed on a person. While acute stress is adaptive and allows the body to respond to its needs, chronic stress has many consequences, one of which is increased probability of headaches [4–6]. Regardless of its form, stress can directly cause an increase in headache frequency [7]. The cyclical association between headaches and stress may be explained through the biological concept of allostatic load. The body responds to stress through hormonal and neural mediators to help maintain physiological stability, or allostasis [8]. Allostasis is an adaptive process to help the body cope with habitual stressors, but, when overwhelmed, allostatic load can occur leading to maladaptive outcomes such as functional and structural alterations of the brain, perceived as headaches [8, 9].

Historically, disease outbreaks have been noted to increase stress in the affected populations. COVID-19 is the colloquial term for Severe Acute Respiratory Syndrome Coronavirus 2 (SARS-CoV-2). COVID-19 first appeared in Wuhan, China in 2019 and progressed into an international pandemic in 2020 [10]. As the virus advanced, health agencies and governments implemented major social changes including mandated quarantines, masking, and increased health measures. COVID-19 is thought to have differentially affected the stress levels of various cohorts [11]. Especially high stress levels were reported in females, people who are single, and those with pre-existing mental health disorders–especially anxiety and depression [12]. While it is true that pandemic-related changes had an impact on everyone, researchers identified university students as a population that was especially affected [13]. In the Philippines, students during COVID-19 reported greater depressive, anxiety, and stress symptoms, and greater overall psychological impact compared to the employed population [14]. Similar results were found in a study conducted in Changzhi, China, where 24.9% of the student participants reported experiencing anxiety because of the outbreak [15]. Factors that contributed to students’ stress included academic delays, reduced social support, and alterations to daily life and routine [16]. Similarly, a group of Spanish researchers found that approximately half of student respondents displayed moderate to severe psychological impact (including depression, anxiety and stress) from the pandemic [17]. In the Spanish study, scores on the Depression Anxiety Stress Scale (DASS-21) and the Impact of Event Scale (IES) suggested that 20–35% of student participants had symptoms of common mental health disorders [17].

Although stress is known to trigger headaches, Delussi et al. (2020) [10] reported that COVID-19 induced quarantine resulted in a reduction of headache symptoms in the first 5–6 weeks of quarantine. A study conducted with pediatric migraineurs presented similar results of decreased headache frequency and intensity during the COVID-19 lockdown period [17]. On the other hand, Al-Hashel and Ismail (2020) found that the COVID-19 pandemic has been accompanied by increased headache frequency, with participants reporting increased stress (a common migraine trigger) and decreased ability to communicate with physicians and receive treatments during lockdown [13]. To our knowledge, no researchers have evaluated how university students’ headache frequency and intensity have been affected by the COVID-19 pandemic.

In the present study, we explored the correlations among levels of general perceived stress, headache debilitation (including headache frequency and intensity), fear of COVID-19, and quality of life. Extrapolating from previous research, we predicted that both general stress and fear of COVID-19 will correlate positively with headache frequency and intensity. In an exploratory capacity, we examined which of the two (i.e., general stress and fear of COVID-19) accounted for more variance in headache debilitation.

## Method

### Participants

We chose the undergraduate students at the University of Saskatchewan as the population in this study. The respondents in this study were over the age of 18 and were recruited from the senior author’s institution (University of Saskatchewan) via its participant pool program. The participant pool system was accessible for all the students at the University of Saskatchewan in participating courses regardless of their college or level of study. No exclusion criteria were set for data collection and all the collected data from completed surveys were processed and analyzed. This study was approved by the University of Saskatchewan’s ethics review board affiliated with the researchers’ department (Psy-REC # 2020-2021-003_HLST). Before accessing the questionnaires, a written online consent form was distributed. This document outlined the purpose of the survey and informed participants of their rights to anonymity, confidentiality, to withdraw from the study at any time without penalty or consequence and to omit any survey items they did not wish to answer. After agreeing with the terms and conditions detailed on the consent form, the participants were able to access the online survey.

This survey was accessible to students in all colleges. No information was collected to indicate which college each participant was in. All the instruments were provided simultaneously through a continuous questionnaire on Surveymonkey. The questionnaire took an average of 24 minutes to complete.

Of 234 respondents, seven individuals were eliminated for not completing the survey. This led to analysis being conducted on 227 participants, of whom 73.6% self-identified as female and 25.1% self-identified as male. Additional demographic information about the participants can be found in Table 1. Responses were collected between January 28, 2021 and February 12, 2021. During this period, the provincial seven-day average of daily new cases of COVID-19 ranged between 170 cases/day and 247 cases/day [18]. In Saskatchewan, the first doses of the COVID-19 vaccine were administered on December 16, 2020 but access to the vaccine was extremely limited for young adults [19].

**Table 1:** Participant demographics.

Characteristic	Value
**Gender [*n (%)]***	
Female	167 (73.6)
Male	57 (25.1)
Other	2 (0.9)
**Age *[Mean (SD)(range)]***	21.29 (4.78) (18–66)
**Year of study [*n (%)]***	
First year	80 (35.2)
Second year	56 (24.7)
Third year	45 (19.8)
Fourth year	27 (11.9)
Fifth year +	19 (8.4)
**Living Arrangement [*n (%)]***	
With Roommate(s)	39 (17.2)
With Family	160 (70.5)
Alone	14 (6.2)
With Spouse	14 (6.2)

### Measures

#### Migraine Disability Assessment Test (MIDAS)

The MIDAS assesses the degree to which migraine headaches impede a person’s ability to perform daily tasks and function normally. It is an internationally validated test and has shown adequate scale score reliability with a Cronbach’s alpha coefficient of .76 in the United States and .73 in the United Kingdom [18, 19]. This instrument is also shown to have a moderately high test-retest reliability ecoefficiency (0.80) [20]. The MIDAS consists of five questions (e.g., On how many days in the last three months did you not do household work because of your headache?) For each single item, participants numerically report the number of days in the last three months that have been affected (e.g., 5 days not doing household work due to headaches). These numbers are summed for all five questions and evaluated based on a scale: a score of 0–5 indicates “Little or No Disability,” 6–10 is “Mild Disability,” 11–20 is “Moderate Disability,” while 21+ indicates “Severe Disability.” In this study, the MIDAS is employed as an indicator of migraine headache frequency and disability.

#### Headache Impact Test (HIT-6)

The HIT-6 is a reliable and valid tool for determining the impact of headaches for both episodic and chronic migraines [21]. Participants answer six questions (e.g., When you have a headache, how often do you wish to lie down?) using the following response format: “Never,” “Rarely,” “Sometimes,” “Very often,” or “Always.” The HIT-6 is quite similar to the MIDAS as it measures how headaches affect the health and well-being of an individual, but it is especially useful in evaluating headache intensity, which is how it was used in this study [22]. In terms of its psychometric properties, scale score reliability for the HIT-6 is consistently between .82 and .90 [21].

#### Perceived Stress Scale (PSS)

The PSS enables individuals to evaluate how stressful their current life conditions are. It consists of 10 questions (e.g., In the last month, how often have you been angered because of things that were outside of your control?) and uses a five-point response format: “Never,” “Almost never,” “Sometimes,” “Fairly Often,” or “Very Often.” For scoring purposes, responses to four positively keyed items (i.e., items 4, 5, 7, and 8) should be reversed. In this sense, higher scores on the PSS denote higher perception of stress in individuals. This scale not only evaluates the existence of situations that one could generally find stressful, but also the stress the respondent might experience because of it. With a Cronbach’s alpha score consistently above 0.70, the PSS has been validated in a variety of populations including people with chronic headaches [23, 24]. This instrument is also shown to be valid for assessing perceived stress among university students [25] and has also been used in headache-related studies [26].

#### COVID Stress Scale (CSS)

The CSS, developed by Taylor et al. (2020) [27], includes 36 items on a five-point scale (0–4) of “Not at all” to “Extremely.” It measures the degree to which COVID-19-related factors have caused individuals stress over the past seven days. Higher scores on this scale indicate higher levels of stress. The CSS consists of six subscales: 1) COVID danger (items 1–6; e.g., I am worried about catching the virus), 2) socio-economic consequences (items 7–12; e.g., I am worried that the grocery stores will close down), 3) xenophobia (items 13–18; e.g., I am worried that foreigners are spreading the virus because they’re not as clean as we are) 4) contamination fear (items 19–24; e.g., I am worried that social distancing is not enough to keep me safe from the virus), 5) traumatic Stress (items 25–30; e.g., I have bad dreams about the virus) 6) compulsive media checking (items 31–36; e.g., I have searched the internet for treatments for COVID-19). The scores for each subscale are summed, with the total overall score denoting the intensity of COVID stress. Cronbach’s alpha scores for each of these subscales have exceeded 0.80 [27], suggesting the measure possesses good scale score reliability. Inclusion of this measure allows for the identification of correlations between COVID-19 induced stress with headaches and/or stress.

#### World Health Organization Quality of Life Scale (WHOQOL-BREF)

This 26-item scale is the brief version of a 100-item scale (WHOQOL) which measures perceived quality of life across four domains: 1) physical (including activities of daily living, dependence on medicinal substances and medical aids, energy and fatigue, mobility, pain and discomfort, sleep and rest, and work capacity), 2) psychological (including body image and appearance, negative feelings, positive feelings, self-esteem, spirituality/religion/personal beliefs, thinking, learning, memory and concentration), 3) social (including personal relationships, social support, and sexual activity), and 4) environmental (including financial resources, freedom, physical safety and security, health and social care, accessibility and quality, home environment, opportunities for acquiring new information and skills, participation in and opportunities for recreation/leisure activities, and the physical environment [e.g., pollution]). It uses a five-point response format (“Not at all,” “A little,” “A moderate amount,” “Very much,” and “Extremely”). This scale requires two forms of score transformation: First, the raw score for each subdomain is converted to a range between 4 and 20 (comparable to the WHOQOL-100) based on the suggested item recoding manual. Second, the new scores are transformed to a 0 to 100 scale. Higher scores denote greater perceived quality of life. Cronbach’s alpha coefficients for the WHOQOL-BREF are consistently above 0.70 [28, 29]. This measurement was shown to be efficient in discriminating between ill and healthy subjects. Test-retest reliability coefficients are .66 for physical, 0.72 for psychological, 0.76 for social relationships and 0.87 for the environment subdomains. This measurement has also been utilized in previous COVID-related studies [30].

As no validated scale currently exists at the time of this study which measures all of stress, headache frequency, headache intensity, quality of life, and impact of COVID-19, the above validated scales were combined to accrue a broad understanding of each participant’s headache, stress, life, and COVID-19 experience.

## Results

This study utilized SPSS 27.0 for statistical analyses. We initially evaluated the scale score reliability of all summative measures using Cronbach’s alpha. All Cronbach alpha coefficients were between .78 and .94, indicating satisfactory to excellent scale score reliability for all scales (Table 2).

**Table 2:** Reliability statistical analysis for the utilized measurements (MIDAS, HIT-6, CSS, PSS, and WHOQOL-BREF).

Instruments	N	Mean	Standard Deviation	Cronbach’s alpha	(95%) Confidence Intervals	
					Lower band	Upper band
MIDAS	227	18.07	21.26	.78	.71	.81
HIT-6	226	57.52	8.19	.87	.84	.89
CSS	227	69.78	20.94	.94	.93	.95
PSS	226	18.43	6.26	.86	.84	.89
WHOQOL-BREF	224	240.23	52.18	.82	.75	.84

The participants’ scores on each scale were compared based on their demographic characteristics such as sex, age and social living arrangement. To compare the differences between male and female scores on each scale, we ran multiple independent samples *t*-tests. Differences were noted on the MIDAS, HIT-6, and CSS, with females reporting greater headache frequency [MIDAS], intensity [HIT-6] and stress about COVID-19 [CSS]. No statistically significant differences between male and female participants were noted for the PSS and WHOQOL-BREF (see Table 3). Our Pearson correlation coefficients did not show any statistically significant associations between participants’ age and their total scale scores. We also examined whether participants’ scores differed in accordance with their social living status (e.g., living alone, with family, with roommates or common-law). Based on results from Kruskal-Wallis *H* tests, no statistically significant differences were observed (see Table 4).

**Table 3:** Independent samples t-tests for the difference in mean scores of male and female participants in the utilized measurements (MIDAS, HIT-6, CSS, PSS, and WHOQOL-BREF).

	Gender	Mean	N	*t*-value	df	P Score	Cohen’s *d*
PSS	Female	19.41±5.90 15.53±6.51	167	4.186	222	< .001***	.62
	Male		57				
HIT-6	Female	58.75±7.62 53.54±8.63	166	4.859	221	< .001***	.63
	Male		57				
CSS	Female	72.13 ±21.44 63.18 ±18.37	167	2.821	222	< .001***	.44
	Male		57				
MIDAS	Female	16.85±15.71 11.96 ±15.34	167	2.024	217	.04***	.31
	Male		57				
WHOQOL-BREF	Female	237.60±51.42 247.84±54.84	164	-1.274	219	0.20	.19
	Male		57				

*** *p* value < 0.05

**Table 4:** Association between age and scale scores (Pearson’s correlation coefficients). Differences in Scale Scores Based on Living Arrangements (Kruskal-Wallis H Test) in the Utilized Measurements (MIDAS, HIT-6, CSS, PSS, and WHOQOL-BREF).

**Scales Based on age**	Pearson’s *r*	Sig		
MIDAS	-.015	.830		
HIT-6	.015	.822		
PSS	-.127	.058		
CSS	-.047	.482		
WHOQOL-BREF	.016	.817		
**Scales Based on Living Status**	H	df	Sig	Eta squared
MIDAS	5.46	3	.14	.024
HIT-6	4.23	3	.23	.013
PSS	.28	3	.96	.004
CSS	.91	3	.82	.018
WHOQOL-BREF	4.46	3	.21	.001

### Bivariate correlation

To identify possible associations among our measures, multiple bivariate Pearson correlation coefficients were conducted. Looking at the total scales, statistically significant positive associations were observed between headache frequency and intensity, and between perceived stress and quality of life (p < .001) (Table 5). Furthermore, perceived stress correlated positively with headache intensity and headache frequency, COVID-19 induced stress correlated positively with perceived stress, and COVID-19 induced stress correlated negatively with quality of life (p < .001). Post hoc power analysis for each correlation and the associated sample size surpassed the recommended .80 threshold in psychological research, indicating that each correlation had sufficient power based on the sample size.

**Table 5:** Pearson’s correlation between the scales of the study (MIDAS, HIT-6, CSS, PSS, and WHOQOL-BREF).

		WHOQOL-BREF	PSS	CSS	HIT-6	MIDAS
WHOQOL-BREF	Pearson Correlation	1				
	Sig. (2-tailed)					
	N	224				
PSS	Pearson Correlation	-.591**	1			
	Sig. (2-tailed)	< .001				
	N	224	219			
CSS	Pearson Correlation	-.316**	.409**	1		
	Sig. (2-tailed)	< .001	< .001			
	N	224	227	220		
HIT-6	Pearson Correlation	-.271**	.455**	.270**	1	
	Sig. (2-tailed)	< .001	< .001	< .001		
	N	224	226	226	226	
MIDAS	Pearson Correlation	-.246**	.431**	.263**	.689**	1
	Sig. (2-tailed)	< .001	< .001	< .001	< .001	
	N	218	221	221	220	227

**. Correlation is significant at the 0.01 level (2-tailed).

### Multiple linear regression

We used multiple linear regression to determine if headache intensity (HIT-6) can be predicted by general perceived stress (PSS), headache frequency (MIDAS), as well the subdomains of COVID-19 induced stress (CSS), and quality of life (WHOQOL-BREF).

No violations for key assumptions pertaining to multiple regression analysis (e.g., presence of outliers, multicollinearity, etc.) were identified. We ran a multiple linear regression with scores on the PSS, MIDAS, four individual subscales of CSS (i.e., danger, socioeconomics, checking, and xenophobia), and four subdomains of WHOQOL-BREF (i.e. physical, psychological, social an environmental) as predictor variables, and scores on the HIT-6 as the outcome variable (Table 6).

**Table 6:** Multiple regression model summary for predicting headache intensity (HIT-6).

Model	R	R Square	Adjusted R Square	Std. Error of the Estimate	F Change	df1	df2	Sig. F Change	Durbin-Watson
1	.601	.362	.325	6.75245	9.914	12	210	.000	2.051

The overall model, *F* (12, 210) = 9.914, was statistically significant (*p* < .001) and accounted for 32.5% of the variance in self-reported headache frequency/intensity (i.e., HIT-6). Standardized beta coefficients revealed that three variables emerged as statistically significant predictors: scores on the MIDAS, scores on the PSS and the scores on the physical subdomain of WHOQOL-BREF (Table 7). Therefore, participants who reported greater migraine disability, greater levels of perceived stress and greater changes in the physical quality of life, were more likely to report experiencing more frequent/intense headaches. Squared semi-partials suggest that the amounts of unique variance accounted for by the MIDAS, the PSS and the physical subdomain of WHOQOL-BREF were 12.1%, 6.3%, and 1.6%, respectively.

**Table 7:** Regression coefficients for headache intensity (HIT-6).

Scale	Unstandardized Coefficients		Standardized Coefficients Beta	*t*	Sig.	Correlations		
	B	Std. Error				Zero-order	Partial	Part
Constant	40.631	4.301		9.447	.001			
MIDAS	.146	.023	.379	6.317	.001	.500	.400	.348
PSS	.472	.104	.353	4.546	.001	.437	.299	.251
WHO (physical)	.111	.048	.178	2.293	.023	-.128	.156	.126
WHO (psychological)	.028	.046	.048	.615	.539	-.150	.042	.034
WHO (social)	-.009	.028	-.024	-.332	.740	-.209	-.023	-.018
WHO (environmental)	-.042	.038	-.082	-1.087	.278	-.246	-.075	-.060
CSS (danger)	-.037	.124	-.026	-.300	.764	.204	-.021	-.017
CSS (Socio-economics)	-.027	.161	-.012	-.168	.867	.188	-.012	-.009
CSS (Checking)	.174	.125	.101	1.394	.165	.258	.096	.077
CSS (traumatic stress)	-.076	.149	-.039	-.508	.612	.219	-.035	-.028
CSS(Xenophobia)	-.008	.130	-.004	-.058	.953	.086	-.004	-.003

## Discussion

Individuals with headache disorders are affected by varying triggers, but stress is evidently one of the most common causes of headaches. Given that life during the COVID pandemic caused shifts in individuals’ physical and mental health condition across ages and life stages [31, 32], we sought to investigate how students’ headache experience is associated with COVID-19 related stress, general stress, and students’ quality of life. We also aimed to discover the predictors for headache characteristics during the COVID-19 transition and investigate differences between male and female students’ headache experience during the pandemic.

As expected, we found a moderate positive correlation between headache intensity and frequency. This finding supports previous literature which identified a positive association between scores on the MIDAS and HIT-6 [20]. One might have predicted that, while headaches were still frequent, intensity could be reduced during the pandemic as staying at home granted those experiencing a headache the ability to treat it instantly (e.g., reduce noise, find a dark space, practice medication, etc.). Our findings support the idea that headache frequency and intensity are correlated, both in pandemic and pre-pandemic times.

Moreover, quality of life correlated negatively with fear of COVID-19, general perceived stress and, to a lesser extent, with headache frequency and intensity. This is an expected finding as excess fear and stress are both negative states and, therefore, decrease the quality of a person’s life. Differing between stress and fear, however, is the directionality of their association with quality of life. It is a logical possibility that stress and quality of life have a bi-directional relationship wherein decreasing one’s quality of life could lead to increased personal stress, or causing a lot of stress to a person results in a reduced life quality. Fears unique to COVID-19 and quality of life may have a more unidirectional relationship. As a person’s fear of COVID-19 increases, their quality of life may decrease. It is less logically compelling to assert that a person with a lower quality of life would, consequently, experience greater COVID-19 related fear.

We identified stronger correlations between general perceived stress (PSS) and headache intensity and frequency than between fear of COVID-19 (CSS) and headache intensity/frequency. This result might have interesting implications for exploring how university students have been affected by the COVID-19 outbreak and university stress in general. COVID-19 caused interruptions in multifarious aspects of daily life routines and academic programs were not an exception. Earlier pandemic studies by Kelloway et al. (2012) [32] and Yip et al. (2010) [33] demonstrated increased stress and decreased mental health (and, therefore, decreased quality of life) during the SARS epidemic. Similar COVID-related papers showed that students have been under increased stress throughout the pandemic [14, 15]. Yet, regarding the idea that those individuals with headache experience could be assumed to be more vulnerable to a wider variety of stress triggers [6, 7], none of these studies had specified whether there is a relationship between students’ physical health conditions and their stress domain levels. We found statistically significant associations between scores on the CSS and scores on the MIDAS and the HIT-6; however, scores on the CSS accounted for incremental variance in headache intensity/frequency above and beyond general perceived stress, quality of life, etc. Our results still indicated that perceived stress was closely associated with students’ headache characteristics during the pandemic.

A key finding was that participants’ scores on the HIT-6 correlated more strongly with general perceived stress than with stress/fear associated with COVID-19. There are several factors that may have contributed to this result. Upon reviewing the content of our stress and fear scales, we noticed that some aspects of university life such as social concerns or fears regarding the lack of normal routines were not highlighted in the COVID-19 fear scale. Therefore, factors in students’ lives that cause stress and were directly impacted by COVID-19 were not specifically measured in our COVID-19 tool, as this tool was created to assess fear caused by the pandemic–not stress. So, while we can suggest that university students’ degree of fear related to COVID-19 is not associated with or predictive of headache experience, we are unable to say the same about COVID-19 related stress. Stressors which were impacted by COVID-19 likely contributed to student stress but were not specifically evaluated on the COVID-19 scale we used. For example, there are reports of increased rates of student workload during the transition to remote learning, which can be regarded as a significant addition to stress in students’ lives [34]. COVID-19 may have also indirectly altered other aspects of students’ life such as restrictions in social opportunities, or student finances, especially through lost job opportunity [35]. Such university student stressors could be considered more general, rather than the CSS items. Due to the aforementioned reasons, we suspect that there are underestimated criteria which could have impact on student stress and headache experience during the pandemic.

As stated, general perceived stress was found to be part of a model which significantly predicted headache intensity. This finding supports previous survey-based research stating that two days of increased stress was strongly predictive of headaches, and the opposite conditions were protective against headaches [36]. Survey research demonstrating that perceived, daily stress is predictive of headaches can help those who commonly experience headaches to better forecast their headaches and adjust behaviors to minimize headache interruptions such as by getting more sleep and incorporating daily relaxation activities. Additionally, this finding may benefit researchers who are interested in brain imaging and understanding the physiological processes that lead to headaches.

We found, unsurprisingly, that quality of life was negatively correlated with headache intensity. While we noticed that the physical domain of our scale for quality of life (WHOQOL-BREF) still had a negative correlation with headache frequency in our study, it showed a positive beta square when explaining headache intensity in the presence of other predictors. The seven questions addressing the physical domain of WHOQOL-BREF include different aspects of life including mobility, physical energy, quality of sleep, work productibility and the ability to keep up with daily routines. While all these aspects were more or less impacted by COVID-19, it is hard to measure the degree of relationship between physical health and headache intensity. Further studies are suggested to investigate the cyclical relationship between different physical health characteristics and different aspects of headache experience, including its frequency and intensity.

We found that females reported higher levels of perceived stress, fear of COVID-19, and headache frequency and intensity, while males reported higher levels of quality of life during the COVID-19 pandemic. It is known that females are more likely to experience headaches relative to males [37], and our research supports this by indicating that, during the pandemic, females had exacerbated levels of headache-inducing factors relative to males during the pandemic. COVID-19 appears to have had greater psychological, economical, and emotional impact on females relative to males as a consequence of multiple social factors [38, 39]. Prior to the pandemic, women were more likely to be occupied by unpaid care work such as child rearing, and house or elderly care [38]. According to Power (2020), the onset of the global pandemic disproportionately increased these demands on women relative to men [38]. This effect was seen across educational and economic spectrums, as Deryungina et al. (2021) [40] noted these effects among female academics (i.e., relative to their male counterparts, female academics with children have seen a larger reduction in research time). School and daycare closures have increased childcare demands, which have fallen heavily on females [39, 40]. Our female participants reported more serious/frequent headaches, more general and COVID-specific stress and, yet, they did not report poorer quality of life can be related to the finding that these factors significantly predict headache intensity. The findings of this study could be used to indicate how the psychological challenges of COVID-19 pandemic, including stress has caused physiological impacts on the university students. Although there is a current optimistic decrease in the COVID-pandemic, we still need to study the impacts of the previous pandemic on the mental health condition of the individuals. As previously highlighted, headache disorders can cause extreme suffering and impact an individual’s ability to function on a daily basis. Relative to how common and debilitating these disorders are, we know little about them. Therefore, increasing our understanding about how large events such as the COVID-19 pandemic impacted individuals with headache disorders, we can attempt to understand how other events may or may not contribute to headaches. While the COVID-19 pandemic was known to be an additional cause of stress for people impacted with prolonged health conditions such as migraines, we focused on specifically investigating how, during the pandemic, general stress and acute and specific stressors (such as COVID-caused stress) could have impacts on headaches and how their perceived headache is influenced by such stress indicators. Migraineurs and those with headache disorders are known to be vulnerable towards mental health disorders such as anxiety and depression [5]. Knowing this information could be helpful with discovering the extent to which those with headache disorders are increasingly vulnerable to stress, mental health conditions, and worsening symptoms, and how, consequently, they are adapting their lifestyle during the pandemic (and other stressful times).

## Future directions

Continuing research relating COVID-19 induced stress to headaches after the pandemic may provide additional insight as to how university student headaches are affected. Re-administering the questionnaire to our study population post-COVID-19 would allow us to compare stress levels to headache intensity and frequency in pandemic and non-pandemic times. Combining the results from the current and prospective study using time-series analysis will reveal temporal differences, and implications may be drawn. Longitudinal effects of the pandemic on student migraineurs also may be observed through this method.

In addition, the insights gleaned from this research may be utilized by physicians treating students with headaches. Through understanding the factors which may be contributing to or reducing an individual’s headache experience, physicians can give targeted advice on lifestyle adjustments most likely to reduce intensity or frequency of headaches.

## Limitations

As one of the main limitations of the present study, we did not collect type of headache and whether the students have been diagnosed with COVID-19. Furthermore, we did not deliver questions that could directly assess students’ stress caused by university-specific factors. These questions may have included subjects such as how the pandemic altered workload, screen time, and social life or dating, and how these changes affected their stress. Obtaining information regarding how the pandemic altered participants’ lives would enrich our results and allow us to more clearly see the impact the COVID-19 pandemic has had on stress levels and headaches. Additionally, it would have been interesting to collect data about what degree each participant was pursuing. Each degree has differing amounts of stress attributed to it, and we may have seen that students in some study areas were more or less impacted by COVID-19 related stress. Furthermore, there was a large discrepancy between the number of male and female participants, limiting their group comparability; having said that, our t-test analysis showed no significant difference in the results based on sex differences. Finally, since we had a quite small sample size in this study (n = 227), caution should be exercised when attempting to generalize the findings of this study.

## Supporting information

S1 File (SAV)
